# Rescuing lymphocytes from HLA-G immunosuppressive effects mediated by the tumor microenvironment

**DOI:** 10.18632/oncotarget.6044

**Published:** 2015-10-09

**Authors:** Danli Wu, Isere Kuiaste, Philippe Moreau, Edgardo Carosella, Patricia Yotnda

**Affiliations:** ^1^ Center for Cell and Gene Therapy, Baylor College of Medicine, Houston Methodist Hospital, Texas Children Hospital, Houston, TX, USA; ^2^ Breast Cancer Center, Baylor College of Medicine, Houston, TX, USA; ^3^ CEA, Institut des Maladies Emergentes et des Thérapies Innovantes (IMETI), Service de Recherches en Hémato-Immunologie (SRHI), Hôpital Saint-Louis, Paris, France; ^4^ Université Paris-Diderot, Sorbonne Paris-Cité, UMR E5, Institut Universitaire d'Hématologie, Hôpital Saint-Louis, Paris, France

**Keywords:** cancers, immunosuppression, HLA-G, ILT2, lymphocytes

## Abstract

Several studies have demonstrated that the antitumor activities of both T and natural killer (NK) effector populations are limited by the immunosuppressive strategies of tumors. In several malignant transformations, the expression of HLA-G by tumor cells rises dramatically, rendering them strongly immunosuppressive. In this study, we postulated that the absence of HLA-G receptors would prevent the immunosuppressive effects of both soluble and membrane-bound HLA-G. Thus, we investigated the therapeutic potential of effector NK cells genetically modified to downregulate the expression of ILT2 (HLA-G receptor) on their cell surfaces. We have shown that the proliferation of modified NK is still dependent on stimulation signals (no malignant transformation). ILT2^−^ NK cells proliferate, migrate, and eliminate HLA-G negative targets cells to the same extent parental NK cells do. However, in the presence of HLA-G positive tumors, ILT2^−^ NK cells exhibit superior proliferation, conjugate formation, degranulation, and killing activities compared to parent NK cells. We tested the effectiveness of ILT2^−^ NK cells *in vivo* using a xenograft cancer model and found that silencing ILT2 rescued their anti-tumor activity.

We believe that combining ILT2^−^ NK cells with existing therapeutic strategies will strengthen the antitumor response in cancer patients.

## INTRODUCTION

Normally expressed in immune privileged sites such as the thymus and trophoblast, HLA-G has been detected on numerous malignant cell types. It is expressed on many blood tumor types including, chronic lymphocytic leukemia, acute myeloid tumor, and multiple myeloma [1, 2], and also on solid tumors like lymphomas [3], melanoma [4], neuroblastoma [5], breast cancer [6], and lung cancer [7]. Preclinical models have shown that the expression of HLA-G on cancer cells renders them more metastatic and significantly decreases survival in mice [8]. Clinical data have shown that HLA-G is associated with poor prognosis when expressed on lymphomas [9], leukemic cells [10], breast cancers [11], glioblastoma [12], and multiple myeloma [13]. Indeed, immunohistochemistry analyses of tumor biopsies and serology have demonstrated that investigating the expression of HLA-G is relevant for determining the prognosis of solid cancers [14]. However, the consensus for liquid tumors has not yet been reached [15-18]. HLA-G lowers antitumor protection through various pathways [19]. After interaction with its main receptor ILT2, HLA-G blocks the activation of NK and cytotoxic T cells as well as their killing activities, conferring the tumor with protection against immune cells [20, 21]. Therefore, HLA-G promotes tumor escape [22-24]. Recently, it has been reported that like tumor cells, unmanipulated MSC also express and secrete HLA-G that confers an advantage to the tumor [25, 26]. Soluble HLA-G not only impairs effector-lymphocyte functions [27], it also increases their expression level of ILT2 and Fas ligand, thus further limiting their efficacy [28]. The efficacy of NK cells is correlated to the level of HLA-G expression on target cells [29]. Clinical studies have shown an association between the downregulation HLA molecules and cancer progression, making NK cells the best choice for immunotherapy targeting resistant cancers. Indeed, infiltrating NK cells are found in advanced cancers, but are not associated with tumor regression, most likely due to immunosuppression by molecules such as HLA-G. We hypothesize that blocking the interaction of HLA-G with its receptor ILT2 would eliminate this immunosuppression and rescue the antitumor activity of NK and other immune cells. In this proof-of-concept study, we investigated the possibility of rescuing such effector cells by knocking down ILT2. Our findings revealed that we have potentiated the antitumor activity of NK cells by extending their killing efficacy to HLA-G^+^ resistant cancer targets. We propose to use these newly armed immune cells as a therapeutic tool to eliminate cancer cells that would otherwise evade the immune system. ILT2-modified NK cells in combined therapies would be beneficial for cancer immunotherapies and could be combined with T-cell therapy to target tumors with heterogeneous levels of MHC class I expression. The use of genetically modified NK cells could be extended to other immune cells and adapted to other human malignancies.

## RESULTS

### HLA-G/ILT2 expression

**Figure 1:**
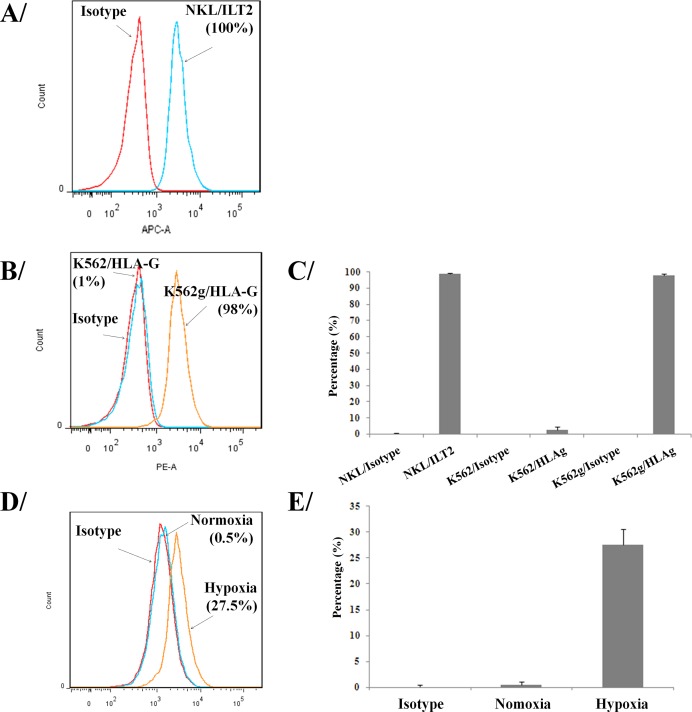
Expression of HLA-G and its ligand on tumor and immune cells Flow cytometry assessment of ILT2 (HLA-G ligand) on NKL cells **A.** and **C.** Flow cytometry analysis of HLA-G surface expression on K562 and modified K562g cells **B.** and **C.**, and HLA-G up-regulation on HLA-G^−^ LCL cells submitted to hypoxia **D.** and **E.** Results shown in A, B, and D are from one representative experiment of four performed. **C.** and **E.** show the percentage of positives cells for 3 pooled experiments in each panel (means ± SD).

We first confirmed by flow cytometry that all NK cells used in our studies express the ILT2 receptor at their surface (Figure 1A and 1C). To investigate the immunosuppressive effects of HLA-G, we have used LCLg cell line [23] and generated a K562g cell line that expressed HLA-G using an in-house lentivector encoding HLA-G1 cDNA. We enriched HLA-G positive tumor cells using a FACSAria (Figure 1B and 1C). Results showed that 98% of transduced K562 cells expressed HLA-G. HLA-G^+^ tumor lines were routinely checked for the expression of HLA-G. As previously reported [30], our results also confirmed that HLA-G^−^ LCL cells exposed to 1% oxygen tension in a hypoxia chamber overexpressed HLA-G (Figure 1D and 1E).

### Validation of ILT2 silencing

**Figure 2:**
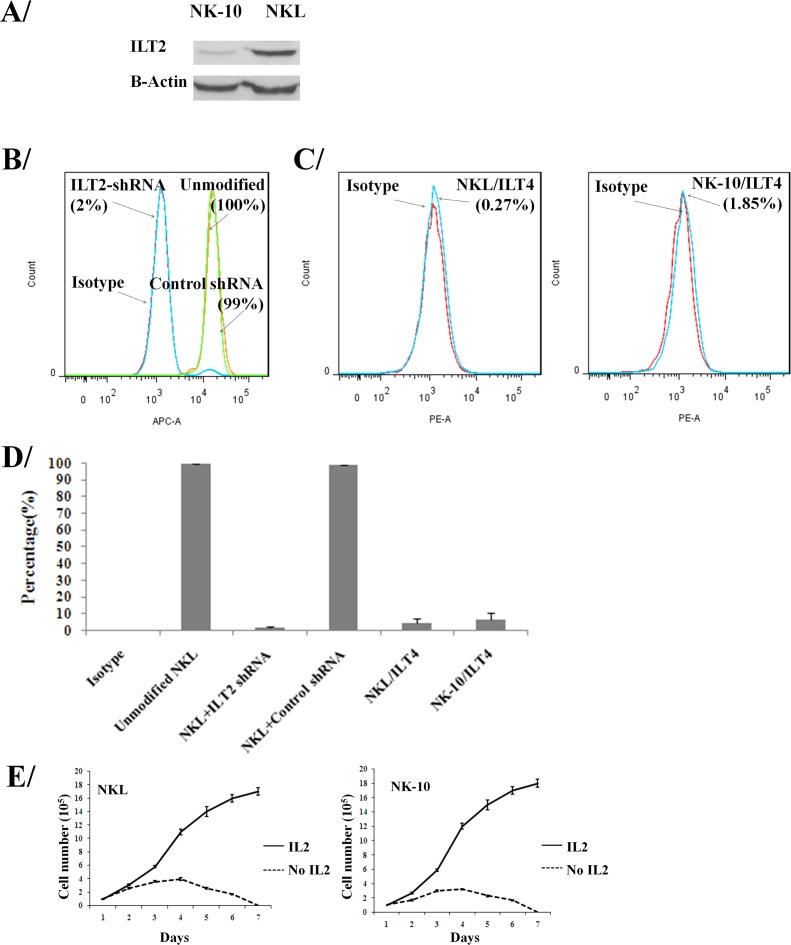
NK cells can be engineered to silence ILT2 ILT2^+^ NKL cells were transduced with ILT2-shRNA and analyzed by western blot **A.** (actin and ILT2 were stained on the same membrane) and flow cytometry **B.** and **D.** for the expression of ILT2 at their cell surface. Flow cytometry analysis for the surface expression of ILT4 on NKL and NK-10 (ILT2^−^ NKL) cells **C.** and **D.** Histograms are from one representative experiment out of four independent experiments performed. Flow results of 3 pooled experiments (means ± SD) for ITL2 and ILT4 staining are shown in **D.**. The viability of NKL and NK-10 (ILT2^−^ NKL) cells cultured with and without rIL-2 was recorded over a period of 7 days **E.**.

To silence ILT2, we transduced ILT2^+^ NKL cells with lentivectors encoding ILT2-shRNA and scrambled-shRNA, amplified and assayed them for ILT2 expression. To obtain a homogenous population, ILT2^−^ NKL cells were sorted by flow cytometry. The cell lysates from parental and modified NKL cells were analyzed by western blot and results showed silencing of ITL2 in modified NKL cells (Figure 2A). Control non-transduced NKL cells, scrambled control-shRNA, and ILT2-shRNA transduced NKL cells were also tested for ILT2 expression by flow cytometry. Results revealed that 100% all parental NKL cells (100%) expressed ILT2; however, only 2% of the ILTL2-shRNA (ILT2^−^ NKL) transduced NK cells expressed this molecule (Figure 2B and 2D). ILT2^−^ NKL line is called NK-10 in our studies. The downregulation of ILT2 in ILT2-shRNA transduced NKL (NK-10) cells demonstrates efficient silencing. ILT2 was equally detected on control-shRNA transduced and unmodified NKL cells (99% and 100%), demonstrating the specificity of ILT2 silencing. Results also showed that less than 2% NK-10 expressed ILT4 (Figure 2C and 2D).

NKL cells closely mimic primary NK cells, since their survival depends on growth factors. To ensure that genetic modifications of NKL cells did not affect their behavior by rendering them independent of growth factors [31], we withdrew recombinant IL-2 (rIL-2) from the cultures and evaluated cell proliferation and viability. In identical culture settings, we supplemented cultures with rIL-2 to obtain proliferation and survival curves. Results showed that NKL and NK-10 (ILT2^−^ NKL) cells proliferate equally well in the presence of rIL-2 (solid line) (Figure 2E). For both cell lines, we observed equal inhibition of cell proliferation and increased cell death following rIL-2 withdrawal (dashed line). These results confirm that the proliferation and the survival of modified NK10 cells still depend on rIL-2.

**Figure 3:**
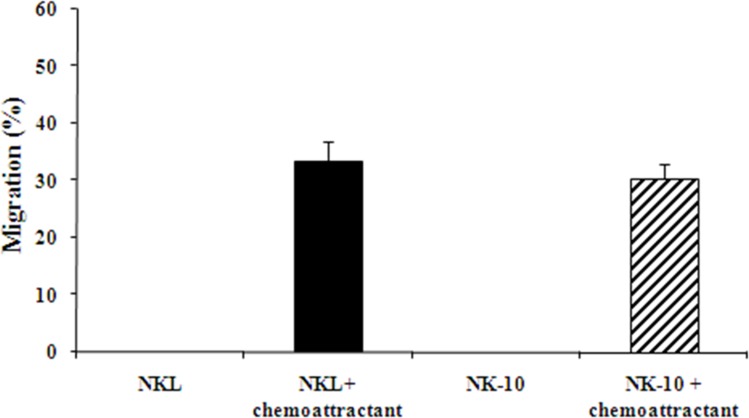
Unimpaired migration activity ILT2-shRNA modified NK cells NKL (black bars) and NK-10 (ILT2^−^ NKL) (striped bars) cells were used in transwell assays to assess their migration with or without chemoattractant. Representative migration analysis shows the percent of cells migrating through the insert in each condition. Data represent the means ± SD of three independent experiments performed.

To ensure that NK-10 cells migrated as well as NKL cells, we used transwell assays. No cell migration was observed in control wells (no chemoattractant) for either NKL or NK-10 cells. We consistently found similar numbers of NKL and NK-10 cells migrating to the lower wells in response to chemoattractant (Figure 3), reflecting the fact that the genetic modification did not avert the ability of NK-10 cells to migrate.

### NK-10 cells display improved antitumor functions against HLA-G^+^ tumors

**Figure 4:**
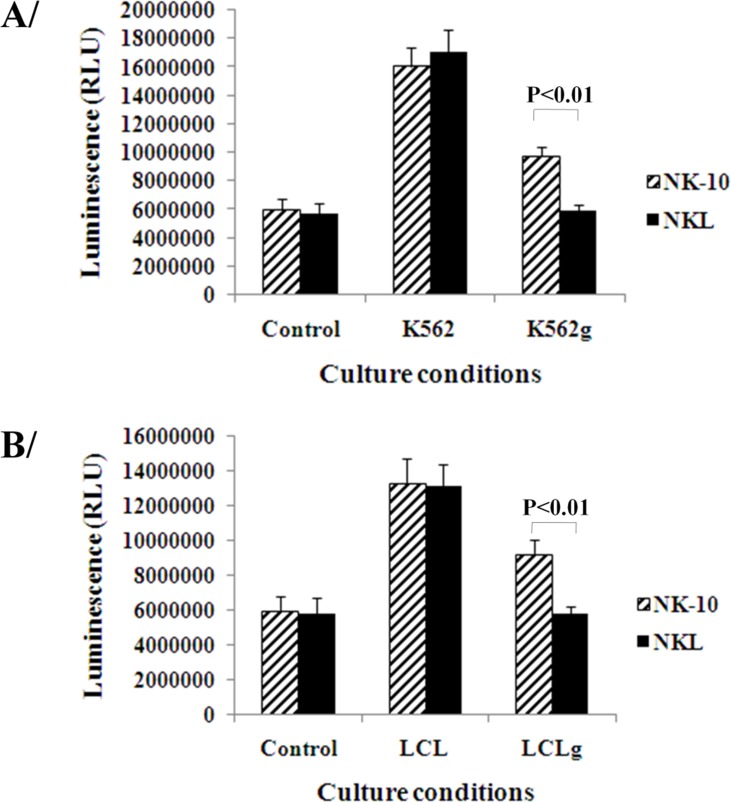
Silencing ILT2 restores the proliferation of NK cells Parental and modified NK cells were co-cultured with tumor cells with or without HLA-G expression (upper panel-A K562, lower panel-B LCL). Proliferation of NK-10 (ILT2^−^ NKL cells) (striped bars) and NKL (black bars) were evaluated using luminescent, ATP-based assays. Data are representative of one out of three independent experiments performed and show the means ± SD, *p* < 0.01 was considered to be significant.

To determine the ability of NK-10 cells to respond to stimuli, we performed proliferation assays on tumor- activated and non-activated NK-10 and NKL cells. The activation was induced using irradiated K562 and K562g, or LCL and LCLg cells. Figure 4 shows that NKL and NK-10 cells proliferated well following cell activation using irradiated K562 or LCL cells. As expected, the proliferation of NKL cells was drastically reduced in the presence of HLA-G-expressing K562g or LCLg cells (Figure 4). Finally, we found that NK-10 cells have an improved proliferative response to HLA-G expressing tumor cells (*p* < 0.01).

**Figure 5:**
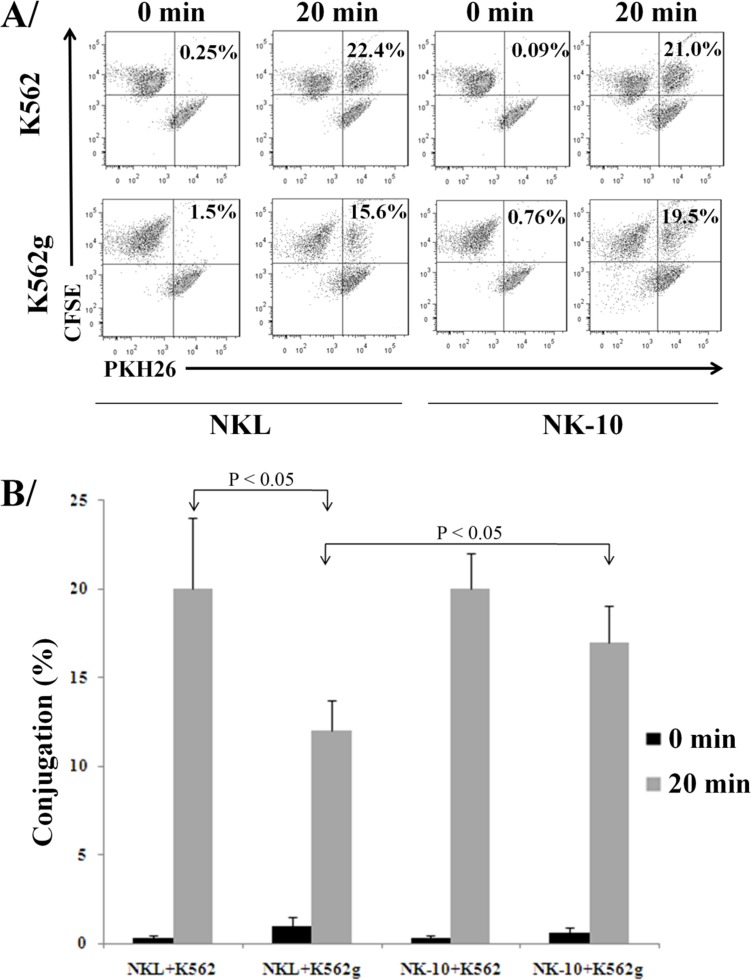
Silencing ILT2 improves conjugate formation in presence of HLA-G Representative dot-plots with double positive signals representing the conjugates formed by NKL or NK-10 (ILT2^−^ NKL cells) and K562 or K562g cells, the percent of conjugates are indicated in each condition **A.**. Percent of effector:targets conjugates obtained with data representing the mean ± SD obtained from 3 independent experiments, *p* < 0.05 **B.**.

In order to kill *via* direct contact, NK cells form conjugates with their targets and secrete lytic granules. We evaluated the killing activity of NK-10 cells using conjugate formation assays, degranulation assays, and killing assays. The formation of conjugates between K652g or K562 cells and NKL or NK-10 cells (stained with PKH26 and CFSE) was evaluated by quantifying *via* flow cytometry the double fluorescent signal (PKH26^+^/CFSE^+^) representative of conjugates (Figure 5A and 5B). Data showed that NKL and NK-10 cells were equally able to form conjugates with K562 cells (22.4% and 21%). When K562g cells were used, however, NKL cells had a reduced amount of conjugates (a decrease from 22.4% to 15.6%, *p* < 0.05) while NK-10 cells produced an almost identical number of conjugates with K562 and K562g cells (21% and 19.5%, respectively).

**Figure 6:**
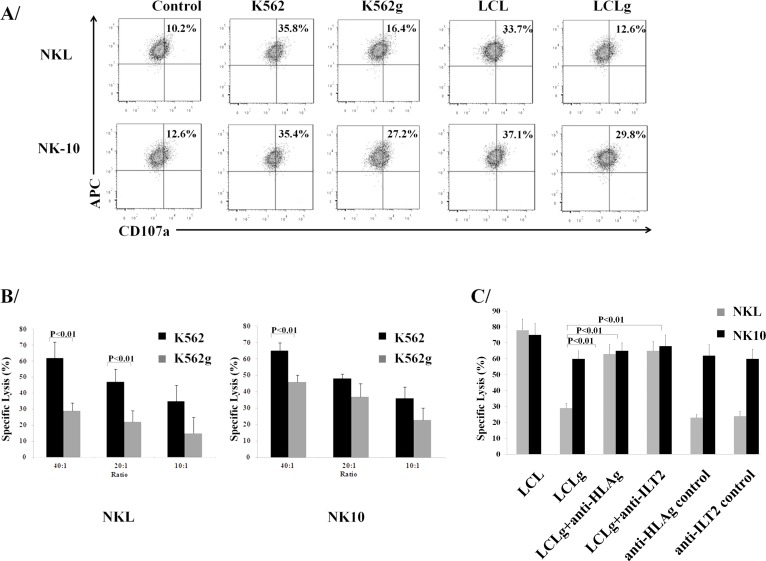
Silencing ILT2 restores the cytotoxic activity NKL and NK-10 (ILT2^−^ NKL cells) co-cultured with K562 and K562g or LCL and LCLg cells were stained with anti-CD107a, anti-CD56, and isotype controls and analyzed by flow cytometry (upper panel) **A.**. K562g cells (grey bars) or K562 cells (black bars) were used to test the killing potential of NKL (bottom left panel) and NK-10 (bottom center panel) **B.**. Comparison of the lytic function of NK-10 cells against LCLg targets with that of NKL cells against blocking anti-ILT2 or anti-HLA-G mAbs-treated LCLg targets (bottom right panel) **C.**. Results shown in **A.** are from one representative experiment out of three performed. Figures **B.** and **C.** show the means ± SD of three independent experiments performed, *p* < 0.01 was considered to be significant.

To investigate the release of lytic granules, NKL and NK-10 cells were co-incubated with K562, K562g, LCL, and LCLg cells, stained for CD56 and analyzed by flow cytometry for the externalization of CD107a. Figure 6A reveals equival degranulation of NKL and NK-10 cells triggered by K562g cells (approximately 35.6%). When NKL cells were incubated with K562g cells, their degranulation decreased by half (from 35% to 16.4%). However, when exposed to K562 cells, NK-10 cells repeatedly exhibited a higher degranulation (27.2%). A similar response was observed with LCL and LCLg; NKL cell degranulation decreased from 33.7% for LCL to 12.6% following incubation with LCLg; while for NK-10 cells, degranulation was higher (37% for LCL and 29.8% for LCLg).

To investigate the restored cytotoxic activity of NK-10, we used LCLg, K562g, LCL, and K562 targets in killing assays. NKL cells kill K562 targets; however, their killing activity decreases in the presence of K562g targets. Compared to NKL cells, NK-10 cells showed an equal efficiency at killing K562 targets. However, these modified effectors demonstrated an improved ability at killing K562g targets compared to NKL cells (Figure 6B, *p* < 0,01). Rouas-Freiss, et al. demonstrated that blocking anti-ILT2 mAbs restore the lytic activity of NK cells [32-34]. Likewise, we performed assays with LCL and LCLg targets, using ILT2 and HLA-G blocking mAbs and their matching mAbs controls. Killing analysis revealed that NKL and NK-10 cells lyse LCL cells to a similar extent, but NK-10 cells exert significantly higher killing activity against LCLg cells. As previously reported, both blocking mAbs restored NKL lytic activity toward LCLg targets (Figure 6C, *p* < 0,01).

### Effect of modified NK-10 cells on HLA-G^+^ tumor growth *in vivo*

**Figure 7:**
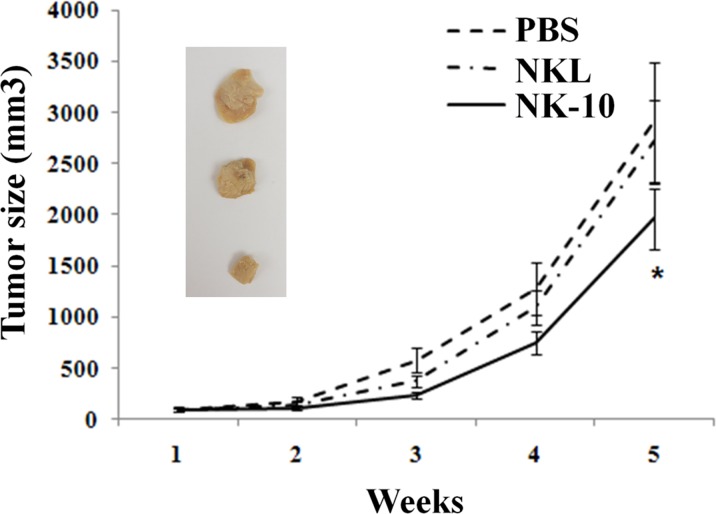
*In vivo* superior efficiency of ILT2 silenced NK effector cells NOD/SCID Mice bearing subcutaneous LCLg tumors (*n* = 8 per groups) were intravenously injected with PBS (dashed line), NKL (Dot-dashed line), or NK-10 cells (solid line). Tumor sizes were measured using a caliper and recorded over time. Data are representative of one out of three independent experiments performed. *p* < 0.05 was considered to be significant. Pictures show representative tumors of each group: PBS (top) NKL (central) and NK10 (ILT2^−^ NKL, bottom).

To test whether, NK-10 cells were resistant to HLA-G immunosuppressive effects *in vivo*, we evaluated their killing activity in a xenograft model. Because of the high proliferation rate of K562 in our *in vivo* model, these tumor cells were not suitable to evaluate the difference between NKL and NK-10 effectors cells. Therefore, we focused our experiment on mice bearing LCLg tumors. NOD/SCID mice bearing LCLg tumors of equal size were infused with either PBS, parental NKL cells (10×106) or NK-10 cells (10×106). Tumor sizes as well as the survival rates of mice from each group were recorded. As expected, results showed that mice receiving PBS exhibited tumor progression. Parental NKL cells had a poor killing effect, as the tumor size of mice receiving these cells was only mildly reduced compared to the tumor size in the PBS group. Mice receiving NK-10 cells (solid line) had smaller tumors compared to PBS (dashed line) and NKL (dot-dashed line) treated mice, reflecting their superior killing efficacy (Figure 7, *p* < 0.05).

## DISCUSSION

HLA-G suppresses key immune cells and is overexpressed in many malignancies [35]. We have shown that HLA-G- tumors can overexpress HLA-G during hypoxia [30], a condition found in resistant tumors. Altogether, these observations emphasize the need to circumvent HLA-G inhibition in cancer immunotherapies.

HLA-G molecules interact mainly with two receptors, ILT2 and ILT4. ILT2 is expressed on most immune cells, and ILT4 is only found on monocytes and dendritic cells. ILT2 binds dimers of α3 HLA-G domains associated with β2-microglobulin (B2M) and ILT4 binds dimers of α3 HLA-G domains free of B2M. Thus cancers that have lost B2M still express both membrane and soluble HLA-G molecules able to interact with ILT4. Membrane HLA-G provides cancer cells a strong protection from immune cells. The release of soluble HLA-G by malignant cells induces a tumor microenvironment that inhibits the antitumor response and promotes tumor progression. KIR2DL4 is only expressed in decidual endometrial NK [36] and its activity and interaction with HLA-G is still controversial [37]. Immune cells exposed to HLA-G^+^ tumor cells overexpress ILT2 at their cell surface, leading to an increased interaction between HLA-G and ILT2, thus further promoting the inhibition of the cytotoxic and allogeneic responses [38]. These findings underline the importance of silencing ILT2 rather than using blocking mAbs to revert HLA-G mediated immunosuppression.

Malignant cells use a wide panel of escape mechanisms to survive immune response, suggesting that each mechanism taken individually is not powerful enough to evade immune response on its own. Therefore, reducing the number of stratagems used by tumor cells could lower the overall impact of tumor immunosuppression. Here, we reasoned that NK cells that lose ILT2 expression would become resistant to HLA-G. Our data support this idea and showed that ILT2 knockdown reestablishes the antitumor potential of NK cells. These results are strongly relevant for immunotherapies when considering the ability of HLA-G to induce a robust inhibition of the immune response; they provide a framework to potentiate cancer immunotherapy protocols.

We first confirmed that our modified tumor cell lines expressed HLA-G and that our NK line expressed its receptor ILT2. We then showed that ILT2-shRNA significantly reduced ILT2 expression at the cell surface of modified NK cells. To our knowledge, this is the first time that ILT2 expression was stably inhibited in lymphocytes. Genetic modification of hematopoietic cells could potentially affect their behavior by regulating the expression of molecules [31] implicated in essential cell functions. NK cells circulate *via* blood vessels and cross tissue barriers to reach primary tumors and metastases. Migration is an important function required to achieve this process. Our results established that silencing ILT2 did not affect the migration potential of NK-10 (ILT2^−^ NK) cells. Similarly, we showed that following ILT2 reduction, cell proliferation was still controlled by cytokines, implying that NK-10 cells would not become malignant.

Triggering ILT2 inhibits lymphocyte proliferation; we expected this function to be rescued in NK-10 cells. We found that NK-10 cells proliferate as well as parental ILT2^+^ NKL cells in non-deleterious conditions (in the absence of the tolerogenic HLA-G signal), again confirming that the genetic modification with shRNA ILT2 did not impair this function. Furthermore, we discovered that modified NK-10 (ILT2^−^ NKL) cells have a significant proliferative advantage over parental ILT2^+^ NKL cells when they were cultured in the presence of HLA-G. This observation, supported by findings from two different cell lines (K562g and LCLg), confirmed that inhibitory molecules will spur parental ILT2^+^ NKL cells into an anergy-like state, but only moderately affect NKL cells devoid of ILT2. We also evaluated the functional relevance of ILT2 silencing in reestablishing the cytotoxic activity of NK cells, using conjugate formation assays, cell-mediated cytotoxic assays, and degranulation assays. We found that ILT2^−^ NK cells have a therapeutic advantage over ILT2^+^ NK cells, as reflected by their substantially increased ability to form conjugates, degranulate, and kill target cells despite the presence of HLA-G. Finally, in line with our *in vitro* results showing rescued antitumor activity for NK-10 cells, we investigated the *in vivo* killing potential of these cells. We found in our xenograft model that NK-10 cells have an increased protective effect compared to their parental counterpart, since treatment with NK-10 cells results in significantly delayed tumor growth in mice. We chose to use low numbers of effector cells in order to adequately detect the inhibitory effect of HLA-G and mimic physiologic situations. The infusion of fewer NK cells could in part explain the moderate therapeutic effect obtained. The absence of tumor regression can also be explained by the high proliferation rate of the tumor cell line and the unfavorable murine environment for human cell persistence, rather than by the tumor escape mechanism.

Additionally, unlike most *in vivo* NK studies which co-inject tumor and NK cells, we injected a single dose of NK cells into mice that already had established tumors, which could also explain the absence of tumor regression. Moreover, we should also take into consideration the rapid growth rate of the xenograft LCLg tumors once established in mice (visible to the naked eye) and the length of time that effector cells injected in the tail vein need to reach the tumor site located in mice flanks (about 5-7 days as determined by IVIS imaging). Unlike their parental counterparts, genetically modified (K562g and LCLg) target cells express some class I molecules at the cell surface that could limit the efficacy of our modified effector cells *in vivo*. This effect is unlikely to occur in a clinical setting, as tumor target cells would not be genetically modified but would express HLA-G endogenously. Finally, engineered NK-10 cells showed a 1.58% increase of the ILT4 receptors expression (delta-MIF of 263 for NKL and 596 for NK-10). In a clinical setting, this small number of cells could be easily removed before reinfusion. Of note, ILT4 is also a receptor for HLA-G but is not expressed on NK and T cells. Thus, the interaction between ILT4 and HLA-G occurs only in the context of monocyte and dendritic cells. Indeed, ILT2 is the main ligand for HLA-G on NK and T lymphocytes. Some events of the immune response may not be replicated in our xenograft model. In physiological situations, NK cells produce pro-inflammatory cytokines and chemokines that attract other immune cells (T cells, dendritic, macrophages, etc …) which further eradicate tumor cells. Tumor cell death itself (even in the absence of complete eradication) would also lead to the production of molecules that increase tumor immune response (cytokine production, antigen release, etc …). Such signals would also recruit other inflammatory cells, thus intensifying the immune response at the tumor site. Therefore, the significant delay of tumor progression mediated by NK-10 (ILT2^−^ NK) lytic activity is relevant and important for cancer immunotherapies. Liang, et al have demonstrated that HLA-G binds PIR-B (homologue of ILT3) expressed on murine dendritic cells and inhibits them [39]. PIR-B is expressed on APC and B lymphocytes, but not on NK or T cells. Thus, its interaction with HLA-G could mimic that of ILT4, but not of ILT2. Furthermore, PIR-B only interacts with dimers of α1-domains [40]. If the ligand for PIR-B were to be identified, it is reasonable to hypothesize that in our mouse model, in addition to HLA-G, PIR-B ligand could also interact with ILT4 expressed on NK-10 cells.

Undeniably, the silencing of ILT2 could raise concerns about the consequences of preventing a “beneficial inhibition” of the immune response. We have previously reported the efficacy of a safety suicide system for genetically modified lymphocytes that could be implemented if needed in this approach to avoid any autoimmune or lymphoproliferative disorders [41].

Because our protective modification occurs directly on effectors in NK cells, our strategy should circumvent the issues of immunogenicity (anti-idiotype Abs), short life *in vivo*, systemic dilution, toxicity to normal tissues (Abs are injected in high doses to circumvent the limitation of the dilution), and bio-distribution (difficulties to reach poor vascularized tumor sites and to cross tissue barrier) often encountered in therapeutic strategies using “blocking antibodies”. Fons, et al. have demonstrated that HLA-G inhibits angiogenesis [42]; therefore, unlike NK cells, therapeutic blocking antibodies would not reach poorly vascularized tumor areas that are aggressive. Of note, the availability of functional mAb is very limited for both HLA-G (only 87G) and ILT2, and existing mAbs are not able to recognize all HLA-G isoforms or different HLA-G dimers.

Other studies have used a preclinical model to investigate the interactions between HLA-G and the anti-tumor response. Lin, et al. correlated the overexpression of HLA-G with the metastatic potential of ovarian cancer cells, as well as with the poor survival of tumor-bearing mice [8]. The same group showed that HLA-G not only impairs NK cells, but it also triggers tumor metastasis *via* the up-regulation of MMP15 [43]. Concomitant to these discoveries, Lee, et al. detected HLA-G in highly proliferative areas of tumor mass in a retinotranplantation model of retinoblastoma [44]. In an ILT2- transgenic mouse model, Liang, et al. demonstrated that ILT2 expression impairs lymphocyte effector function and allows the survival of fully allogeneic skin graft *in vivo* [45]. Favier, et al. established the ability of infused HLA-G recombinant proteins to trigger ILT2 on effector cells and favor skin engraftment in a murine *in vivo* transplantation model [40]. Finally, Agaugué, et al. investigated HLA-G mediated tumor escape in an immunocompetent murine model of melanoma and revealed that specific mAb-blocking HLA-G reverts its ability to promote tumor cell growth. They demonstrated that human tumor cells expressing HLA-G grow in an immunocompetent host by affecting both innate and adaptive immunity through expansion of myeloid-derived suppressive cells, loss of peripheral T cells, and cytokinic balance in favor of Th2 (increased IL-4, IL-10, and IL-13) over Th1/Th17 cytokines. Th2 *versus* Th1/Th17 constitute the main mechanisms by which HLA-G promotes tumor expansion [46]. The findings of these studies clearly underlined the importance of HLA-G in tumor progression, metastasis, and as an immune escape mechanism. Our future studies further examining the role of silencing ILT2 using primary NK and T cells should confirm the efficacy of our approach.

In conclusion, we explored the potential of blocking ILT2/HLA-G interaction to protect immune cells and restore their antitumor activities. Indeed, we found that stably silencing ILT2 alleviates the inhibitory effect of HLA-G on many vital functions, reestablishes the immune activities of NK cells and increases their antitumor response. These findings have significant implications for the design of more robust cancer immunotherapies for which silenced-ILT2 NK cells could be combined with other therapies to tackle more immune escape mechanisms. Similarly, therapies that directly target tumor cells could be combined to silenced-ILT2 NK cells to boost the overall antitumor effect and benefit cancer patients. These results prove informative not only for patients with cancer but also for other diseases (such as HIV [47]), where the presence of HLA-G prevents patients’ recovery. ILT2 is also expressed on B cells, and HLA-G has been found to inhibit their proliferation, differentiation, and Abs production. Thus, our findings would also have an application for autoimmune diseases in which HLA-G plays an important role, as demonstrated in Multiple Sclerosis [48] and in coeliac diseases [49].

## MATERIALS AND METHODS

### Cells

Human embryonic kidney (HEK) 293T cells (ATCC) were cultured in DMEM (Mediatech Inc, Manassas, VA). K562 cell lines (ATCC) and K562g expressing HLA-G membrane-bound proteins (HLA-G1 isoform) were generated in our laboratory. EBV transformed B lymphoblastoid (LCL), LCL 721.221 (LCL), LCL 721.221 cells expressing HLA-G (LCLg), and NKL cells (human NK leukemia, strictly depends on IL-2 for survival) were kindly provided by E. Carosella. NKL, K562 and LCL lines were cultured in RPMI-1640 (Mediatech Inc). All media were supplemented with 100 U/ml penicillin, 100 μg/ml streptomycin, 250 ng/ml Amphotericin B, 2 mM L-glutamine, and 10% fetal calf serum (Hyclone, Logan, UT). For hypoxia cultures, we used a hypoxia chamber filled with 1% oxygen [50].

### Vector preparation and transduction

Lentivectors containing the short hairpin RNA (shRNA) targeting ILT2 and control scramble shRNA were prepared using 293T cells. The cells were transfected with the lentiviral plasmids and the accessory plasmids (VSV-G, gag, pol and rev genes) (pLKO.1, OpenBiosystem). Virus preparations were concentrated and cells were spinfected in the presence of 8 μg/ml polybrene (Sigma-Aldrich, St. Louis, MO). To increase transduction efficiency, cells were retransduced with the same vector. Control (scramble RNA) shRNA lentivector was used to obtain the threshold of ILT2 expression against which specific silencing was measured.

### Immunoblot analysis

Effector cells were lysed and electrophoresed on SDS-polyacrylamide gels, then immunoblotted with anti-ILT2 mAb and anti-actin. After washes, the blots were incubated with secondary HRP-antibodies at room temperature. Immunolabeling was detected by ECL (Amersham Pharmacia Biotech).

### Apoptosis assays

Briefly, cells were stained with FITC-Annexin-V and 7-Aminoactinomycin D (7AAD) or propidium iodide (PI) and analyzed by flow cytometry using a FACSAria flow cytometer.

### Proliferation assays

Co-culture proliferation assays were performed using effector cells (2×10^3^) incubated with γ-irradiated (40 Gy) tumor cells (1×10^3^, expressing HLA-G or not) at a 2:1 ratio. Controls consisted of NKL cells or NK-10 cells alone. Proliferation was assessed using an ATP assay (CellTiter-Glo G7570; Promega, Madison, WI) and a luminometer, or by Trypan blue. In the IL-2 withdrawal tests, viable cells were counted under a microscope.

### Cytotoxicity assays

Effector cells were activated with 200 U/ml of rIL-2. Target cells were loaded with calcein (Sigma-Aldrich, St. Louis, MO) for 1 h at 37°C, then washed. Effector cells were then mixed with 1×104 target cells at ratios of 10:1, 20:1 and 40:1 and incubated for 4 h at 37°C. The maximum calcein release was obtained with target cells incubated with 2% Triton X-100; calcein spontaneous release was measured using target cells incubated in assay media only. Supernatants were analyzed using a Spectrofluorometer (SpectraMax Gemini). The percentage of specific lysis was calculated using the formula: [(test release - spontaneous release)/(maximum release - spontaneous release)] x 100. For blocking assays, Fc receptors were blocked then the cells incubated for 30 minutes with blocking anti-HLA-G (Exbio, Prague, Czech Republic), blocking anti-ILT2 (Becton Dickinson), or their control mAbs used at 10μg/ml[23].

### Degranulation assays

NK cells were prepared as for cytotoxic assays but used unstained. Effector cells were then mixed with 1×10^4^ target cells at a ratio of 10:1. CD107a mAb or isotype control was added. Plates were then incubated for 4 h at 37°C, after the first hour monensin (Golgi stop) was added to the wells. Brefeldin A was added to the wells. After incubation, mAb specific for CD56 or isotype control was added and incubated for 30 minutes in the dark. Cells were analyzed by flow cytometry. Effector and targets cells alone were used as controls.

### Conjugate assays

Effector and target cells were stained with PHK26 (Sigma-Aldrich) and CFSE (Thermo Scientific) dyes, respectively. They were then washed, mixed in a 1:1 ratio, and incubated at 37°C for 0 and 20 minutes. Conjugate formation was analyzed by flow cytometry.

### Migration/chemotaxis assays

Cell migration was assayed in 24-well plates using transwell chambers with a 5 μm pore membrane (Millipore Corp, Billerica, MA). Effector cells were starved and then added to the inserts (10^5^ per insert). Conditioned media containing attractants (serum) or control media were added to the wells. Plates were incubated at 37°C for 4 h. Migrating cells were counted using a hemocytometer.

### *In vivo* assays

NOD/SCID (non-obese, severe combined immunodeficient/beige) mice were maintained according to our Institutional Animal Care and Use Committee guidance, and all procedures were approved by BCM review committee. Six- to eight-week-old mice were sub-lethally irradiated and subcutaneously inoculated with LCLg cells (2×10^6^) in their flank. Mice with tumors of the same size were pooled (*n* = 8 mice per group): Mice received an intravenous injection of either phosphate buffered saline (PBS), NKL alone (10×10^6^), or NK-10 cells (10×10^6^). Recombinant IL-2 and IL-15 were administered twice a week. Tumor sizes were measured with calipers. *In vivo* experiments were performed at least 3 times.

### Statistical analysis

Results reported are from at least three independently repeated experiments. For statistical analysis, Student's *t* test was used. For each test, p values of < 0.05 were considered statistically significant.
